# The complete mitochondrial genome of *Cochylidia moguntiana* (Rössler, 1864) (Lepidoptera: Tortricidae)

**DOI:** 10.1080/23802359.2021.1975514

**Published:** 2021-09-22

**Authors:** Yu Zhao, Yinghui Sun, Yongling Sun, Xiuling Zhang, Zhaobin Xu, Xueli He, Zhoujie Qi, Ying Wang

**Affiliations:** a College of Life Sciences, Dezhou University, Dezhou, Shandong, China; b College of Vocational Education, Dezhou University, Dezhou, Shandong, China

**Keywords:** *Cochylidia moguntiana*, mitochondrial genome, phylogeny

## Abstract

The complete mitogenome of *Cochylidia moguntiana* (Rössler, 1864) was sequenced and analyzed. The genome is 15,433 bp long with a high A + T content (80.6%), and consists of 13 protein-coding genes, 22 tRNA genes, 2 rRNA genes, and a noncoding control region. A phylogenetic analysis of 18 tortricid species for which mitogenes are available showed strong support for the monophyly of Tortricinae.

Since the complete mitochondrial genome of the smaller tea tortrix, *Adoxophyes honmai* was reported in 2006, 20 complete mitochondrial genomes of Tortricidae have been published (Lee et al. 2006; Qi et al. 2021). The first complete mitochondrial genome of the tribe Cochylini (Tortricidae), that of *Cochylimorpha cultana* (Lederer, 1855), was only recently reported by Qi et al. (2021). We sequenced the mitochondrial genome of *C. moguntiana* (Lederer, 1855), a second species of the tribe Cochylini, which provided additional molecular data for an analyses of phylogenetic and evolutionary relationships within Tortricidae.

*Cochylidia moguntiana* is a member of one of the largest genera in the tribe Cochylini (Tortricidae: Tortricinae). A recent molecular phylogenetic analysis of the tribe (Brown et al. 2019) placed *Cochylimorpha* as sister to *Eugnosta* Hübner, [1825] 1816). At present, *Cochylimorpha* comprises 97 species worldwide (Gilligan et al. 2018), with greatest species richness in the Palearctic – China, Russia, and Europe in particular (Sun and Li 2013). The larvae of *Cochylimorpha* utilize mainly *Artemisia* species (Asteraceae), feeding on the seeds, stems, and roots (Razowski 1987). Numerous species of *Cochylimorpha* (or *Artemisia*?) are found in open, dry biotopes, including the steppes (Razowski 2009).

An adult male of *C. moguntiana* was collected in Funing County, Hebei Province, China (39.50°N, 119.26°E) in 2018 by light trap. The specimen was identified based on the text and images in Razowski (1970) and Sun and Li (2012). Total genomic DNA was extracted from the muscle tissues of the legs of the adult using the DNeasy Blood and Tissue kit (QIAGEN Sciences, Valencia, CA, USA). The DNA and voucher specimen of *C. moguntiana* are deposited in the Insect Collection, College of Life Sciences, Dezhou University, Shandong, China (Yinghui Sun, sunyinghui8789@126.com), under the accession code DZU002. COI of *C. moguntiana* (GenBank accession: MM17263) was used as bait to extract the mitogenome of *C. moguntiana*. The genomic DNA was subsequently pooled with that of other insect species and sequenced using the Illumina Nova6000 (PE150, Illumina, San Diego, CA, USA) platform at Novogene Co., Ltd. (Beijing, China). The software IDBA-1.1.1 (Peng et al. 2012) was employed to assemble the data, with similarity set at 0.98. The mitogenome of *C. moguntiana* was then extracted using a Blast search (Altschul et al. 1990) with COI as the bait sequence (Crampton-Platt et al. 2015), and the percentage of identical matches was 100%. The mitogenome annotation was conducted using the methods of Zheng et al. (2020).

Mitochondrial genomes of 18 additional Tortricinae species available from GenBank were used for a phylogenetic analysis. The sequences were concatenated with alignments of 13 PCGs using the default settings in MAFFT (Katoh and Standley 2013). Maximum-likelihood (ML) reconstruction was performed using IQ-TREE (Nguyen et al. 2015) with 1000 bootstraps replicates and the PMSF acid substitution model. Two species of Olethreutinae, *Cydia pomonella* (L.) (GenBank accession No. JX407107) and *Grapholita molesta* (Busck) (GenBank accession No. HQ392511), were used as outgroups.

The mitogenome of *C. moguntiana* is 15,433 bp in length (GenBank accession No. MW413307), containing 13 protein-coding genes (PCGs), 2 ribosomal RNA genes, 22 transfer RNA genes, and one noncoding control region. The overall nucleotide composition was 40.8% A, 39.8% T, 11.4% C, and 8.0% G, with and A + T content of 80.6%. Most of the 13 PCGs have ATN as the start codon (ATA for *ND6*; ATC for *ATP8*; ATG for *ATP6*, *COX2*, *COX3*, *CytB*, *ND1*, *ND4* and *ND4L*; ATT for *ND2*, *ND3* and *ND5*; CGA for *COX1*). The stop codon TAA is assgned to *ATP6*, *ATP8*, *COX3*, *CytB*, *ND1*, *ND2*, *ND3*, *ND4L*, *ND6*, whereas a single T is assigned to *COX1*, *COX2*, *ND4* and *ND5*.

The phylogeny based on the 18 mitogenes from GenBank and our mitogene sequence of *C. moguntiana,* supports the monophyly of Tortricinae (Figure 1). Although the combined sequence data do not represent a broad cross-section of the subfamily, the results of the analysis are highly consistent with that of other phylogenetic analyses of the family Tortricidae (e.g. Regier et al. 2012).

**Figure 1.**
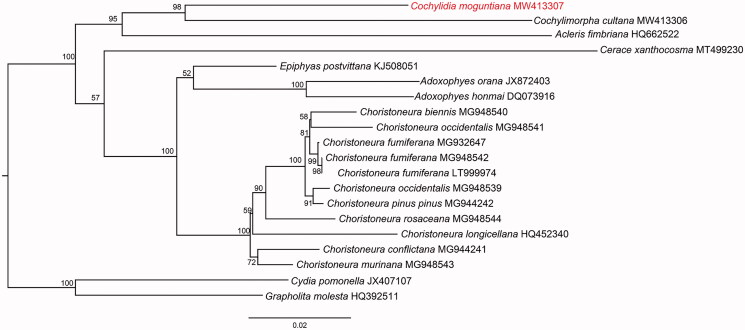
Phylogenetic tree of 18 Tortricinae species and two Olethreutinae species based on the concatenated dataset of 13 PCGs using the maximum-likelihood (ML) method. The alphanumeric terms following species names indicate the GenBank accession numbers.

## Data Availability

The data that support the findings of this study are openly available in GenBank of NCBI at https://www.ncbi.nlm.nih.gov/ under the accession No. MW413307. The associated BioProject, SRA, and Bio-Sample numbers are PRJNA731101, SRR14598117, SAMN19249501, respectively.
